# Progress in Veterinary Medicine in Its Relation to Public Health1A paper read before the New Jersey Sanitary Association at Lakewood, December 7, 1901.

**Published:** 1902-02

**Authors:** William Herbert Lowe

**Affiliations:** Paterson, N. J., President of the Veterinary Medical Association of New Jersey


### PROGRESS IN VETERINARY MEDICINE IN ITS RELATION TO PUBLIC HEALTH.[1]

By William Herbert Lowe, D.V.S.,
PATERSON, N. J.,
PRESIDENT OF THE VETERINARY MEDICAL ASSOCIATION OF NEW JERSEY.

The progress made in veterinary medicine in its relation to hygiene, as well as in many other respects, during the last few years has been constant and amazing, and shows that the two great branches of medical science—human and animal—are inseparable. This fact, I believe, is recognized by scientists the world over. The leading veterinary colleges of America are no longer independent institutions, but have become departments of great universities. Cornell and the University of Pennsylvania have each a veterinary department, and the New York University has recently established a veterinary department. This department is the result of the union of the two oldest veterinary colleges in this country, viz. : the New York College of Veterinary Surgeons with the American Veterinary College and the adoption of the consolidated institution as a school of the New York University, under the title of the New York-American Veterinary College.

Comparative anatomy, comparative physiology, comparative pathology, bacteriology, entomology, animal parasites and parasitism, chemistry, physics, meat- and milk-inspection, and sanitary science are among the subjects found in the curricula of the recognized veterinary schools of to-day. Harvey's discovery of the action of the heart and the circulation of the blood by experiments

[1] A paper read before the New Jersey Sanitary Association at Lakewood, December 7, 1901.

made upon living animals, and Jenner's discovery of vaccination and the announcement that smallpox might be prevented by inoculation with the virus of cowpox, are well-known examples of the inestimable value to mankind of a scientific study of animal life in connection with experimentation. In fact, it might be said that it was by the student entering the domain of comparative or veterinary medicine that many of the now known scientific facts were discovered and demonstrated; and as many of the diseases of animals are transmissible to man, the subject assumes an importance of no small proportion when considered from the stand-point of hygiene or public health. Well, indeed, may the New Jersey Sanitary Association give consideration to this subject.

By investigations and experiments conducted by veterinarians in Texas fever of cattle it was found that cattle in the infected district carried in their blood the contagion of Texas fever; that it was due to a protozoan organism, *pyrosoma bigeminum*, analogous to the parasite of human malaria, and that the parasite was transferred to susceptible cattle outside the infected district by the Southern cattle tick, *boophilis bovis*. The experiments alluded to are interesting to the scientific investigator, and make a remarkable chapter in the progress of medical science. They have already led to extensive studies of the part played by insects in the propagation of human diseases, and particularly malarial fevers, opening up a new field in medical research by which it was discovered that mosquitoes are responsible for spreading malaria.

No fact has drawn a closer relation between animal and human diseases or the protection of the public health through domestic animals than the introduction of the antitoxin principles of serum-therapy. The immunizing of animals against fatal contagious diseases; the rapid and certain diagnosis of latent diseases, which cannot be known by physical symptoms, and their early isolation before they have spread their disease germs among their kind and to the human family; the cure of some diseases by the administration of repeated doses of attenuated virus, and the prevention of development of disease by their early administration, are phases of veterinary science which make it a great preserver of the public health. Contemplate for a moment the importance of tuberculin used for the diagnosis of tuberculosis in dairy cattle; mallein used for the diagnosis of latent glanders in the horse; vaccine employed to protect mankind from smallpox; tetanus antitoxin employed in the treatment of lockjaw; diphtheria antitoxin employed in the treatment of diphtheria, as well as other antitoxins employed in the treatment of anthrax, rabies, and other diseases. It is impossible to estimate the value of animal serums to mankind employed for diagnostic, prophylactic, and therapeutic purposes.

The position taken by Professor Koch in the recent British Congress on Tuberculosis has awakened a great deal of interest in the question of the transmissibility of the tubercle bacillus from animal to man and from man to animal. The experiments showing the difficulty or the impossibility of transmitting human tuberculosis to cattle in a fatal form cannot be accepted as evidence that the bovine bacillus, which is far more virulent and fatal for many animals, cannot infect man. On the contrary, the greater disease-producing power of the bovine bacillus would appear to make it more dangerous for human beings as well as for lower animals. The evidence showing the practical impossibility of cattle being infected with human tuberculosis under ordinary conditions is a great encouragement for the eradication of bovine tuberculosis, since it proves that the danger, so often feared, that cattle if freed from the disease would be immediately reinfected from mankind, does not exist in fact, and need not be considered. Even if it were true that bovine tuberculosis is not communicable to man, it would nevertheless be to the interest of cattle and swine growers to have the disease eradicated as an economic measure; and such eradication would also be to the interest of the public, since it would have great effect as a means of preserving the food-supply of the nation and of protecting consumers from the unwholesome products of diseased animals.

The communicability of tuberculosis received its full share of attention at the recent annual meeting of the American Veterinary Medical Association, held at Atlantic City last September, and resolutions were adopted expressing the sense of the Association as above. I might state that a number of veterinarians are making a series of experiments at the present time that promise to be of no little value to science and mankind. Some of these experiments, I am glad to say, are being made in our own State by the Public Health Committee of the Veterinary Medical Association of New Jersey.

The last Legislature enacted a pure food law, and put the execution of the law under the control of the State Board of Health; and I hope that ample provision is made in said law for necessary veterinary inspection of all food animals as well as animal food products. The qualified veterinarian is the expert on this subject. This Association can do much to enlighten the general public on veterinary hygiene and sanitation, and I regard the same as an important function of this organization.

All dairies should be regularly inspected by a competent veterinarian. The cattle should be examined as to their health, and the attendants as to their own healthfulness and cleanliness; the stables should have sufficient air space, be well lighted, ventilated, and drained; the quantity and kind of food is important, and the water-supply should be uncontaminated. Milk is often tainted, contaminated, or infected after it is drawn from the cow. The whole system of producing, handling, and marketing milk is being revolutionized, and great improvement has already been made in many dairies by enterprising producers. Milk is pure while in the udder of a healthy cow, but the moment exposed, impurities soon deteriorate it. It should be conveyed to the milk-house as soon as drawn. Care must be taken that the water is not polluted in which the milk cans and utensils are washed, for it is in this way that epidemics of typhoid are sometimes started. No dairyman should give medicine to a cow and allow the milk to be used, especially for children. The use of various preparations of mercury, arsenic, zinc, iron, iodine, purgative and other medicines easily cause fatal results.

I am of the opinion that practitioners of human medicine might in certain maladies of their patients, especially of children, administer medicine to their patients through the agency of a cow by her milk.

The importance of meat-inspection is becoming more evident every day. A layman or butcher is not qualified to do work of this character. It requires the services of the veterinary expert. The danger is not so much from meat of an inferior quality, or even from putrid meat, as it is from fresh meat containing micro-organisms of communicable diseases. Certain parasites (*cysticercus cellulosæ* and *C. bovis*) are directly transmissible to man through the use of meat. The veterinarian with a knowledge of these worms is able to prevent the spread of their tapeworm stage among human beings by condemning the diseased meat or subjecting it to processes which will render it harmless. The rigid system of meat-inspection in Germany has resulted in an actual decrease in tapeworm disease (by *tænia solium* and probably also by *T. saginata*) in man and in the frequency of *C. cellulosæ* in the human eye.

Condemnation and destruction of organs infected with certain other parasites (*echinococcus, cœnurus, cysticercus tenuicollis*) will prevent the spread of those parasites in their tapeworm stage to dogs, and by that means prevent the reinfection of man (by *echinococcus*) and of domesticated animals (by *echinococcus, cœnurus, cysticercus tenuicollis*). In this case prevention of tapeworm disease in dogs, though of comparatively little importance so far as the dogs are concerned, becomes very important not only in public hygiene (in the prevention of disease in man and animals), but also from an economical stand-point, preventing financial loss to stock-raisers from disease and death in their herds and flocks caused by these worms. The destruction of livers heavily infected with flukes will also result indirectly in decreasing fluke diseases in man and animals. I would refer those interested to an exhaustive work on the subject compiled by the Bureau of Animal Industry, some of the data of which I have incorporated in this paper.

Pork before being put on the market should be examined microscopically for a minute worm called *trichina spiralis*, which causes the disease known as trichinosis. As a secondary precaution no pork should be eaten unless it has been thoroughly cooked throughout, so as to kill any parasites which may exist in it. The pernicious and most unsanitary practice of feeding swine with garbage should be prohibited, as it induces disease in that animal whose carcass constitutes a large portion of our food resources.

Cold storage, cooking, or salting will destroy the parasites of certain diseases. In other cases the meat should be "tanked" and converted into fertilizer. In each city and town the slaughtering should all be done at one abattoir, and that abattoir should be constructed upon sanitary principles. There should be a competent veterinary inspector appointed as director in every slaughter-house, with assistants if necessary.

Veterinary inspection of meat is now being efficiently carried out on a large scale for interstate and foreign trade by the United States Bureau of Animal Industry. Meat-inspection should be carried out along the same lines by every city and town in New Jersey. The State, and every city and township board of health, should have at least one veterinarian officially connected with it. Veterinarians should be appointed on boards of health as well as physicians and others, for no small part of preventive medicine and sanitary science belong essentially to the veterinary profession.

I take a deep interest in this Association, because it brings together members of several professions interested in a common cause.

